# Non-territorial GPS-tagged golden eagles *Aquila chrysaetos* at two Scottish wind farms: Avoidance influenced by preferred habitat distribution, wind speed and blade motion status

**DOI:** 10.1371/journal.pone.0254159

**Published:** 2021-08-05

**Authors:** Alan H. Fielding, David Anderson, Stuart Benn, Roy Dennis, Matthew Geary, Ewan Weston, D. Philip Whitfield

**Affiliations:** 1 Natural Research Ltd, Aberdeenshire, United Kingdom; 2 Forestry and Land Scotland, Aberfoyle, United Kingdom; 3 RSPB Scotland, Inverness, United Kingdom; 4 Roy Dennis Wildlife Foundation, Forres, United Kingdom; 5 Department of Biological Sciences, University of Chester, Chester, United Kingdom; Centro de Investigacion Cientifica y de Educacion Superior de Ensenada, MEXICO

## Abstract

Wind farms can have two broad potential adverse effects on birds via antagonistic processes: displacement from the vicinity of turbines (avoidance), or death through collision with rotating turbine blades. These effects may not be mutually exclusive. Using detailed data from 99 turbines at two wind farms in central Scotland and thousands of GPS-telemetry data from dispersing golden eagles, we tested three hypotheses. Before-and-after-operation analyses supported the hypothesis of avoidance: displacement was reduced at turbine locations in more preferred habitat and with more preferred habitat nearby. After-operation analyses (i.e. from the period when turbines were operational) showed that at higher wind speeds and in highly preferred habitat eagles were less wary of turbines with motionless blades: rejecting our second hypothesis. Our third hypothesis was supported, since at higher wind speeds eagles flew closer to operational turbines; especially–once more–turbines in more preferred habitat. After operation, eagles effectively abandoned inner turbine locations, and flight line records close to rotor blades were rare. While our study indicated that whole-wind farm functional habitat loss through avoidance was the substantial adverse impact, we make recommendations on future wind farm design to minimise collision risk further. These largely entail developers avoiding outer turbine locations which are in and surrounded by swathes of preferred habitat. Our study illustrates the insights which detailed case studies of large raptors at wind farms can bring and emphasises that the balance between avoidance and collision can have several influences.

## Introduction

Adverse effects of wind farms on birds largely compromise two antagonistic processes which may not be mutually exclusive [1]: displacement from turbines or wind farms through ‘disturbance’ or ‘wariness/fearfulness’, termed meso- or macro-avoidance, respectively [2]; or collision with rotating turbine blades [3]. On the potential absence of mutual exclusivity, red-tailed hawk *Buteo jamaicensis* declined after a wind farm’s construction (inferring avoidance) [4], but there were also collision victims. Similar results have been found for migrating black kite *Milvus migrans* [5,6]. There are other species, such as turkey vulture *Cathartes aura* and raven *Corvus corax*, which appear abundant within wind farms but with very low collision rates (e.g. [7–10]). Several other studies have also commented on how intra-species’ abundance within wind farms do not correlate with recorded strike rates [3,6,11,12; although see 13]. These findings probably relate more to ‘micro-avoidance’ [2]: the capacity to avoid collision with turbine blades once birds have entered turbine arrays [3,14–24].

Staying away from individual turbine locations (meso-avoidance) or entire wind farms (macro-avoidance) can create functional habitat loss [1,2,5,25–28], whereas not staying away can create fatality through collision (even if risk is reduced through micro-avoidance) [1–3,10]. Potential impacts of wind farms thereby can be radically different so far as their planning under ornithological impact assessments [6]. It is important that the predominant process behind birds’ responses to turbines is identified, and why it may vary.

As concerns grow on potential population-level impacts on raptors and other long-lived birds (e.g. [29–32]), measures to mitigate or compensate for adverse effects of wind farms are increasingly being proposed and developed; primarily to prevent or minimise collision mortality or compensate for/offset its impact (e.g. [3,33,34]). One such measure involves Turbine Shutdown Systems (TSSs) or “Shutdown on Demand” systems [3,35]. As birds approach moving rotor blades their rotation can be stopped in anticipation of potential collision event(s). TSSs may be entirely automated (e.g. [36]) or involve more manual procedures (e.g. [37]). TSSs assume that the primary effect to be mitigated is collision risk and birds are less likely to be hit by or fly into a stationary blade than a moving blade [35–42].

Underlying topography can affect the way in which anabatic and/or orographic processes provide external wind energy sources as uplift [43–46]. Soaring birds on migration may exploit different uplift sources to when settled on wintering and breeding grounds, or to resident birds [37,44,47,48]. Consequently, reactions to turbines may differ by annual cycle stage or residency status [2,3,42].

The reaction of flying birds to stationary or mobile rotor blades has rarely been examined explicitly in the context of avoidance. Studies describing flying birds’ approaches to turbines typically do not discriminate rotor blades’ motion status (e.g. [19,49,50]). Early Californian observations speculated higher raptor collision risk at small lattice turbine towers by facilitating perching opportunities [51]. In subsequent observations, however, perching almost always occurred at lattice tower turbines that were inoperative [9,52,53] and inoperative lattice turbines were relatively frequent [54]. This would infer reduced wariness of turbines with stationary rotor blades, although collisions were more likely adjacent to inoperative turbines [55].

Walker et al. [56] remarked that a resident pair of golden eagles *Aquila chrysaetos* in Scotland did not differentiate between stationary or moving blades when avoiding turbines. Records of GPS-tagged Scottish golden eagles during dispersal also showed avoidance and did not differ in proximity to turbines of different rotor diameter, inferring that birds reacted to the presence of turbine towers and not to their blades [57].

As facultative or obligate dependents on external wind energy to fuel flight, soaring birds’ flight behaviour is also often conditional on weather [12,19,58], especially wind speed and direction for those exploiting orographic uplifts from topographic features [12,44–46,48]. Focus via specific turbines on relationships between wind, topography, birds’ flight behaviour and collision risk may be highly informative [8–10,37,48,54,55]. Inadequate orographic wind energy through low wind speed placed an obligate soaring species, griffon vulture *Gyps fulvus*, at greater collision risk by reduced uplift to fly over turbine blades [48].

Numerous studies of birds’ interactions with wind farms involve a single or a small number of wind farms (e.g. [4,47,49,59–62]). Research may effectively treat wind farms as uniform features (e.g. [63,64]). However, when examined, an increasing finding is that wind farms are not homogenous entities, and individual turbines may produce adverse impacts not shown by others in the same facility [8,10,12,37,47,54,55,65,66]. Such findings can be instrumental in future wind farm design or in repowering schemes [6,48,66,67].

Thus, case studies have merit, especially if they involve detailed data which allow focus at fine scales e.g. at specific turbines and/or involving known individual birds’ flight behaviour. Generating detailed data on birds’ flight behaviour increasingly involves the accuracy and precision provided by GPS telemetry [5,18,24], and can be informative even when not collected at operational wind farms [44,45,68,69]. Case studies can thereby complement research at larger scales [68,70–76].

Much research on birds and wind turbines has focused on raptors, especially large soaring species [77]. These species are typically considered to be vulnerable to collision with turbine blades [3,10,77–79] and are sensitive to additive mortality in older individuals [32,53,80,81]. As a facultative-soaring large raptor the golden eagle exemplifies these features of concern. Several studies of golden eagles indicate or assume that birds are not displaced from wind turbines i.e. do not show meso- or macro-avoidance, and so are vulnerable to collision mortality [22,32,34,44,71–73,82–87] although see [43,72]. Results from Scotland are contrary, in substantially finding macro-avoidance [56,57,88,89].

Our study involved two wind farms in central Scotland utilising data from individual turbines on date of first operation and, after operation date: rotor blades’ motion status (stationary or moving) and wind speed. Numerous telemetry records from several dispersing golden eagles tagged with GPS-tags were used to document tag location data or flight line location data on 3-D distances to turbine locations before and after individual turbines’ operation; and after operation, according to blades’ motion status and wind speed. The influence of intrinsic eagle habitat preferences underlying turbine locations and telemetry records was also involved.

Following previous Scottish studies (see above), our first hypothesis was that golden eagles would be further from locations after turbines had begun operating there i.e., golden eagles showed avoidance. Under a second hypothesis, from [56,57], we predicted that eagle flights would not differ in proximity to turbines according to blades’ motion status (stationary/moving). Our third hypothesis expected that eagles would be further from turbines at lower wind speeds because gaining access to turbines’ relatively high elevations was conditional on the orographic uplift energy provided by wind. A contrary alternative hypothesis, following [48], would expect that at lower wind speeds golden eagles would fly closer to operational turbines.

## Methods

### Study area and species

**Fig 1:**
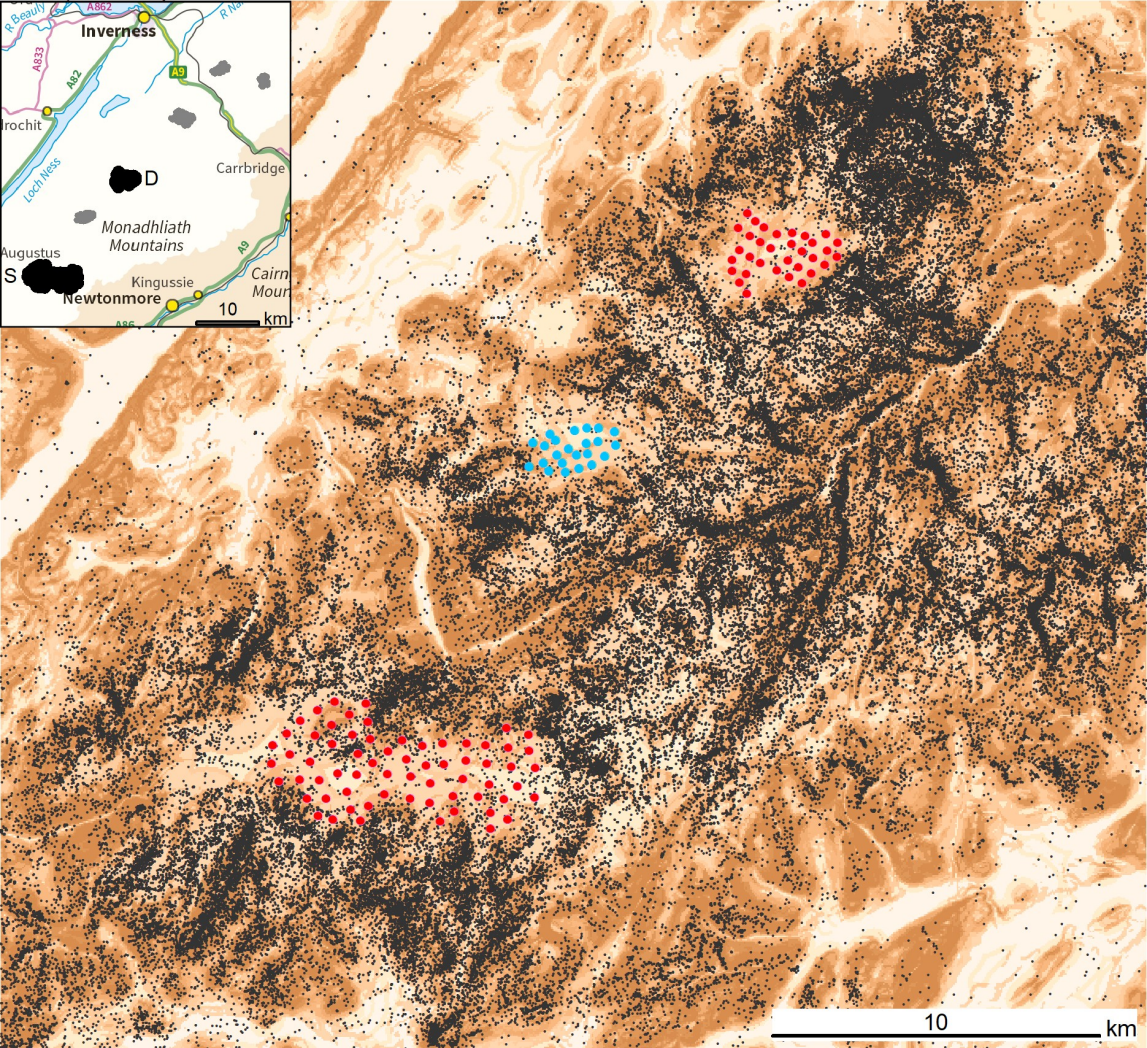
The location of Dunmaglass and Stronelairg wind farms in central Scotland (D and S in black, with other wind farms in dark grey: Internal box). Wider illustration shows the location of turbines (red circles) including the Corriegarth Wind Farm (blue circles), between Dunmaglass and Stronelairg, which was fully operational 30 September 2016. Black dots show non-territorial golden eagle GPS-telemetry records (≈ 530,000) from after Stronelairg became operational in 2018 (see main text). The backdrop is a colour scale representation of eagle habitat preference according to the Golden Eagle Topography (GET) model [45], with darker tone indicating greater predicted preference. Contains Ordnance Survey data © Crown copyright and database right 2017.

Dunmaglass (57.243°, -4.262°) and Stronelairg (57.097°, -4.437°) wind farms are in the Monadhliath Mountains of Scotland’s central Highlands (Fig 1). Dunmaglass has 33 (3 MW) turbines with 80 m towers and three 40 m blades. Main construction works began 1 June 2014; the first turbines were erected May 2016, and turbines were operational between 7 November 2016 and 26 March 2017. Mean elevation at the base of towers is 697 m asl. Stronelairg has 66 (3.6 MW) turbines with 73 m towers and three 57.5 m blades. Main construction works began 20 April 2017; turbines were erected between 21 September 2017 and 5 October 2018 and were operational between 26 March 2018 and 6 November 2018. Mean elevation at the base of towers is 669 m asl.

The vegetation in and around the wind farms is open upland: primarily heather *Calluna vulgaris* moorland, and wet heath. The topography is sloped valley sides of varying steepness cut by watercourses primarily running southwest to northeast, with higher plateaux and smooth ridges at 450–805 m asl. Land use primarily involves the shooting of red (willow) grouse *Lagopus lapopus scoticus* and red deer *Cervus elaphus*.

The study area and the wider region are popular with non-territorial golden eagles during dispersal [90, S. Benn unpublished data]; and see Fig 1. Over the last decade the surrounding wider region has seen a major expansion in occupied territories where breeding productivity is relatively high [90]: much of this expansion has been due to a relaxation in illegal persecution which was associated with intensive management for driven shoots of red grouse in the region [90]; see also [80,89,91]. This expansion has apparently been unimpeded by the presence of wind farms (S. Benn unpublished data). Judging from wider dietary studies [92] and food item collections at nest sites in the region [93] the main diet involves mountain hare *Lepus timidus* and red grouse, with red deer carrion likely common in the non-breeding season. Scotland hosts more than 500 golden eagle pairs occupying territories [94] and substantially more non-territorial birds [89,91].

### GPS-tags and deployment

Eagles were GPS-tagged as nestlings when 50–70 days old, as judged by plumage [95,96] and weighed between 3.4 and 5.0 kg at tagging. Transmitters were fitted using a harness of 13 mm Teflon Ribbon (Bally Ribbon Mills, Bally, PA, USA) using a ‘X harness method’, otherwise described as a “crossover wing harness” [97]. Harnesses had a breakaway feature by stitching through ribbons with either cotton or linen thread at the central point over the sternum ([98,99] intended to remain attached for the minimum expectation of a 3–5 year natal dispersal period [92,100,101]).

Two tag models were deployed:

- 70 g solar powered GPS/GSM transmitters (PTTs) (n = 9, deployment years 2015–2018) manufactured by MTI (Microwave Telemetry Inc., Columbia, MD, USA). Transmission is over the mobile phone (GSM) network and GPS fix rate is dependent on battery charge (dynamic adjusted fix rate dependent on battery charge from 1 per minute to 1 every 2 hours). Transmissions are attempted to GSM network twice daily. Longevity of transmitters was suggested at ≥ 3 years by MTI.
- 70 g solar powered GPS/Argos transmitters (PTTs) (n = 14, deployment years 2007–2015). GPS fixes and transmissions cycles adjusted by pre-programmed fix rate and transmission schedule (duty cycle): maximum fix rate was hourly during daylight hours. Longevity of transmitters was suggested at ≥ 3 years by MTI.

Transmitter weights and harnesses were less than the 3% lower recommended maximum of body weight [102] and the higher recommendation of 4% [99] (see also [103]). All birds were tagged under appropriate licences granted by Scottish Natural Heritage (SNH) and the British Trust for Ornithology (BTO). Satellite tagging should not have adverse effects on study individuals [103,104]. No evidence of adverse effects of tagging has been found in Scottish studies, under physiological, behavioural or demographic evaluations [89].

### Wind turbine data

Data on a per-turbine basis were obtained from wind farms’ operators up to 24 April 2020: date of erection, date of operation, rotor speed, and wind speed (via hub-sited anemometers). Wind speed and rotor speed data were simultaneously available every 3 h for each operational turbine. Data on rotor speed were binary-classified into still (stationary) or moving blades for analyses.

### GPS-tag data

Accuracy of GPS PTT tag location records is given by MTI as ± 18 m horizontally and ± 22 m vertically but can be more accurate in practice [89,105]. Records involve varying HDOP (Horizontal Dilution of Precision) and VDOP (Vertical Dilution of Precision) values per fix. Use of lower values enhances data precision and accuracy. Conservatively we used only fixes < 3.5 HDOP (*cf* < 10 HDOP [106]) in keeping with the 50-m pixel resolution of our habitat preference predictions [46] and see later. We also excluded records if altitude data were unavailable or potentially inaccurate (> 6 km). Records between sunset and sunrise were removed because it was assumed birds would then be roosting and interactions with turbines were improbable. We used the R suncalc library (0.5.0) to estimate sunrise and sunset for each record location and date.

Telemetry records included only those during natal dispersal (or juvenile dispersal: [107–109]: hence, after dispersal from the natal territory [109,110] and before breeding territory settlement [46] (see also Fig 1). Taking the centre of Stronelairg as the ‘destination’ location, the 23 tagged birds originated from nests 5–216 km (mean 67 km) distant; to Dunmaglass centre, natal nests were 15–233 km (mean 71 km) away.

Examination of the influence of turbines on flight behaviour may be confounded if birds’ records are taken from distances where turbines could not possibly be influential [28]. Therefore, analysed data included those fixes or flight line records (see below) within 1 km of a wind turbine location: this cut-off was a precautionary maximum based on previous studies of Scottish golden eagles’ displacement and disturbance distances [56,88,111].

In before-and-after (pre- v post-) operation analyses we used data provided by fix locations (n = 19,629) to estimate distance from a turbine. If a tag record was earlier than the date at which a turbine became operational, or the turbine was under construction, it was assigned to a pre-operational (before) class. Later tag data were assigned to a post-operational (after) class. Availability of tag records, according to wind farm construction and turbine operation dates (see above) meant that for both wind farms many pre-operational telemetry data were during construction. Disturbance in the construction phase may displace birds [63,88]. In pre- and post-operation comparisons for testing the macro-avoidance hypothesis, this made our test conservative.

For post-operation analyses, we used data only from GPS/GSM tagged birds (n = 7 birds after excluding two with < 10 records post-operation). The high temporal frequency of fixes from GPS/GSM tags (see above; [105]) allowed flight lines to be approximated between consecutive records. The accuracy of such approximations depends on the time between consecutive fixes, and all consecutive records > 5 min apart were excluded. The derived flight line could indicate greater proximity to a turbine location than its composite start-end points alone by estimating the shortest orthogonal distance between a flight line and turbine hub.

### Distance to turbines

We used closest distance from a tag record (before-and-after-operation analyses) or flight line (after-operation analyses) to a turbine hub location as our measure of eagles’ proximity to a turbine. Flying birds can avoid wind turbines in 3-D (e.g. [5,43]) and if 2-D horizontal distances are used (e.g. [64]) a bird flying ‘across’ but well above a turbine array could incorrectly be deemed to be close to a turbine location, falsely suggesting no avoidance. Telemetry records’ closest distances to turbine hubs were consequently calculated in 2- and 3-dimensions. The 3-D distance was derived via trigonometry using the closest 2-D distance and the difference in above ground altitudes between telemetry record and turbine hub (80 m and 73 m for Dunmaglass and Stronelairg turbines, respectively). The altitude for a flight line, at its closest point to a turbine, was given by the altitudes of its composite consecutive start and end records weighted by the relative length of the line at its closest 2-D pass location. Approximating the flight segment as a straight line does not allow for micro-avoidance of turbines so distances to turbines were likely to be conservatively low.

Once the closest distance of a flight line to a turbine hub had been calculated, the recorded time (based on start and end fixes) was rounded to the nearest 3 h to match the wind speed and the blade status (stationary/moving) records from the same turbine (see above). The temporally coincidental wind speed and blade status data were used in post-operation analyses.

Most distances to the nearest turbine were to outer turbines. There were few records within the wind farms (< 5% of post-operational location records: Results) and a proportion of these would have been closest to an external turbine. Although it was possible, but sometimes arbitrary (Fig 1), to allocate turbines to an internal or external location this was undesirable as birds’ distances to inner turbines were inevitably skewed by strong right-censorship, especially in 2-D, due to spacing distances between turbines e.g., at Stronelairg the maximum 2-D distance to inner turbines was 250 m while for outer turbines it was the 1,000 m buffer limit.

### Intrinsic habitat preference

Turbine locations may be avoided or not used because they are not in habitat (including air space) preferred by golden eagles and hence we included a measure of habitat preference in analyses. We used the Golden Eagle Topography (GET) model [46] to predict space use by golden eagles independent of the presence of turbines. GET provides a topographically based surrogate for the availability of orographic winds, which have repeatedly been found as influential in habitat selection studies of golden eagle and other large facultative/obligate soaring raptors [46]. ‘GET scores’ range from 1–10 and a GET 6 score is a switch point in preference, so that GET 6+ indicates increasingly preferred habitat. Because a GET score has three elements it is possible, for example, for a GET 6+ score to involve a relatively low preference for altitude, but relatively high preference for slope and distance to ridge. For the background landscape, all 50-m pixels in Scotland had GET scores [46].

A GET score for each turbine location, and hence the indicative eagle preference, was derived from the mode of the 50-m pixel containing the turbine tower and the four surrounding pixels. From tag records, the GET score was also calculated from the 50-m pixel underlying the closest 3-D distance of a tag fix or flight line to a turbine location.

### Statistical analyses

In before-and-after-operation analyses we used turbine location GET score, the tag location GET score, and pre-post operational status as predictors (fixed effects) of the 3-D distance between tag record locations and turbine locations. Our data contained repeated records from the same wind farms, turbines and birds and these were likely to violate the assumption of independent *y*-values [112,113]. Therefore, we used linear mixed models (GLMMs) with wind farm identity, turbine identity and bird identity as random effects in all models. With the involvement of interaction terms, we fitted twelve potential candidate models including a null model with random effects but no fixed effects. We fitted two-way interactions to only those models containing two main effects.

In after-operation analyses we used turbine location GET score, flight line GET score, blade motion status (stationary/moving), and wind speed at turbine hub as predictors (fixed effects) of the 3-D distance between flight lines and turbine hubs. GLMMs included wind farm identity, turbine identity and bird identity as random effects. With inclusion of interaction terms and a null model, 27 candidate models were fitted.

Lowest Akaike’s Information Criterion (AIC) and Bayesian Information Criterion (BIC) scores were used to select the best model from candidate model sets. The best candidate models were assessed by examinations of standardised residuals against fitted values [114]. Modelling used the lmer function (REML = FALSE to obtain AIC and BIC values) from the lme4 package (1.1–26), in program R (3.6.1) [115,116] and the lmerTest (3.1–3) to obtain p values for the best model. We divided the amount of observed variation explained by the best model into a marginal coefficient of determination (variance attributable to the fixed factors), and a conditional coefficient of determination which includes the variance attributable to both fixed and random factors [117]. Partial effect plots were produced using the effects package (4.2–0).

## Results

### Before-and-after-operation analyses

Observed mean distance to a turbine hub was 75 m larger on average once turbines became operational (mean ± sd, CI 95%, n: before 604 ± 273 m, 152–995 m, 2,194; after 679 ± 233 m, 293–990 m, 17,340). Despite the larger turbines, tag records were closer, on average, to the Stronelairg turbine hubs both before and after they became operational (mean ± sd, CI 95%, n: Stronelairg before 599 ± 272 m, 152–994 m, 1,979; Stronelairg after 659 ± 204 m, 270–968 m, 10,203; Dunmaglass before 654 ± 277 m, 151–999 m, 215; Dunmaglass after 707 ± 198 m, 380–697 m, 7,137).

**Fig 2:**
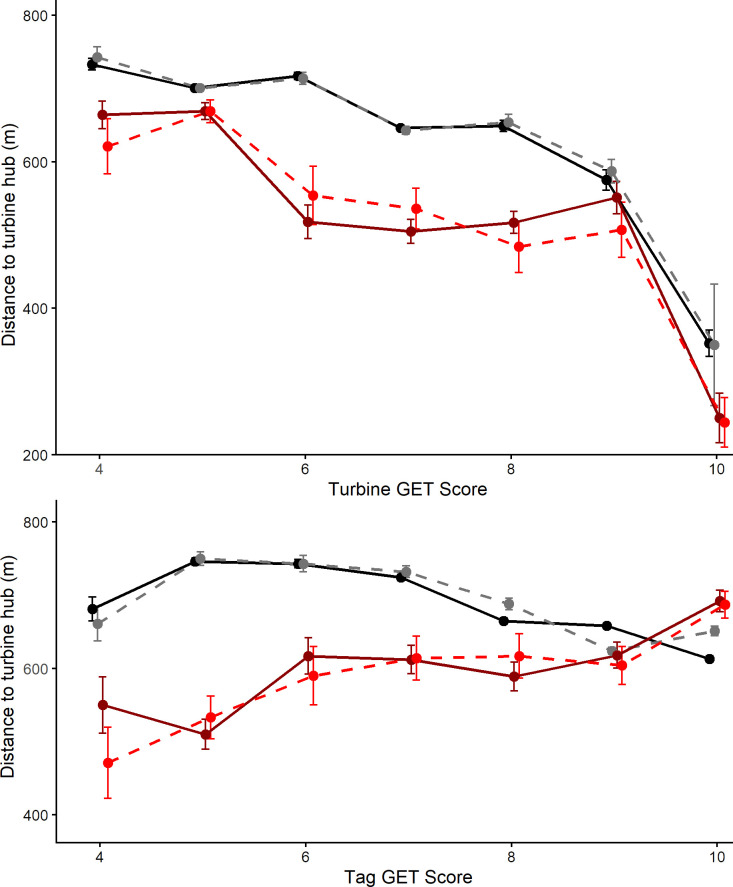
Mean distances to turbine hubs, with 95% confidence limits, in relation to the operational state (before and after). Solid lines are fitted distances from the best model, dashed lines are observed distances. Black and gray lines are post-operation distances while red and dark red lines are before-operation distances. The upper plot is the GET score at the turbine location and the lower plot is the GET score at the birds’ locations.

As the GET score at the turbine location increased golden eagle records were closer (Fig 2) but mean distances were greater after turbines became operational. The relationships between GET scores and distance to a turbine were more complex when the GET score at bird locations were analysed (Fig 2). Before turbines became operational birds were further away at the higher GET scores. A similar relationship was less clear after turbines became operational. Generally, birds were further from the turbines once they became operational except at the higher GET scores. The observed and fitted distances from the best model (see below) were very similar (Fig 2).

In simple descriptive statistics, only one of 17,346 post-operation records was within one rotor blade diameter distance of a turbine hub (< 0.01%) and 83 were within two rotor diameters distance of a turbine hub (0.48%). By comparison, in 2,283 pre-operation records, ten (0.44%) distances were within one diameter distance of a future turbine location and 233 (10.21%) within two diameters distance. Hence, after turbines’ operation, golden eagles were less likely to be recorded ‘close’ to turbine locations and close proximity to operational turbines was rare. Using ΔAIC and ΔBIC [114,118] Model 11, the most parsimonious model was the saturated one, with two and three-way interactions between the three fixed effects (Table 1).

**Table 1:** The twelve model candidates in before-and-after-operation GLMMs with resultant model selection values of ΔAIC and ΔBIC.

Model	Fixed factors	df	ΔAIC	ΔBIC
0	Null model	5	2259.3	2298.7
1	GET score at turbine (GETturb)	6	2261.0	2308.2
2	Pre-post operational status (Postop)	6	2257.1	2304.3
3	GET score at tag location (GETtag)	6	961.3	1008.5
4	GETturb + Postop	7	2258.7	2313.8
5	GETturb + Postop + GETturb*Postop	8	2260.0	2323.0
6	GETturb + GETtag	7	960.0	1015.2
7	GETturb + GETtag + GETturb*GETtag	8	359.0	422.1
8	Postop + GETtag	7	960.3	1015.5
9	Postop + GETtag + Postop*GETtag	7	960.3	1015.5
10	GETturb + Postop + GETtag	8	959.0	1022.0
11	GETturb + Postop + GETtag + GETturb*Postop + GETturb*GETtag + Postop*GETtag + GETturb*Postop*GETtag	12	0	0

(see Table 2 for model 11 AIC & BIC values).

All terms in the best model were highly significant, with the exception of the intercept and the two-way interaction between operational state and the tag GET score (Table 2). No problems were apparent with the residuals. 61.3% of the variance was explained by the model (Table 2); 6.3%, by the fixed factors and 55.0% by the random factors. Therefore, the fixed factors did little to explain the effect on distance, while the identity of the eagles, turbine identity and wind farm showed a strong effect. The largest component of the explained variance was the turbine identity random factor (71.7%).

**Table 2:** Summary statistics for the best GLMM (Model 11: Table 1) examining 3-D distance of eagle tag records to the vicinity of a turbine location in before-and-after-operation analyses: Turbine GET = GET score at the turbine location, Postop = a binary value around the date when a turbine became operational, Tag GET = GET score at the tag location, * indicates interaction.

	Null Model		Full Model	
*Coefficient*	*Estimates*	*P-Value*	*Estimates*	*P-Value*
Intercept	483.96 (398.11 – 569.81)	<0.001	-34.71 (-253.51 – 184.10)	0.756
Turbine GET			76.64 (41.36 – 111.91)	<0.001
Operational (After)			-189.92 (-326.41 – -53.44)	0.006
Tag GET			68.20 (50.91 – 85.48)	<0.001
Turbine GET * Operational			86.80 (62.61 – 110.98)	<0.001
Turbine * Tag GET			-10.04 (-12.99 – -7.10)	<0.001
Tag GET * Operational			11.71 (-5.56 – 28.98)	0.184
Turbine GET * Operational * Tag GET			-9.13 (-12.12 – -6.15)	<0.001
**Random Effects**				
σ^2^	30,626.0		27,247.2	
τ_00_	28,645.2 _T_ID_		30,898.9 _T_ID_	
	5,796.5 _ID_		5,244.8 _ID_	
	2,554.0 _windfarm_		2,481.9 _windfarm_	
N	2 _windfarm_		2 _windfarm_	
	96 _T_ID_		96 _T_ID_	
	23 _ID_		23 _ID_	
Observations	19,534		19,534	
Marginal R^2^/Conditional R^2^	0.000/0.547		0.063/0.613	

Wind farm identity, turbine identity (T_ID) and bird identity (ID) were random factors. 95% CI are given below the estimates. The lower part of the table is information on the random effects. The R^2^ values are marginal (variance of the fixed effects) and conditional R-squared (variance of the fixed and random effects) statistics, based on [117].

The displacement response to turbines was complex, as indicated by the significant interaction terms (Table 2). The level of displacement depended on absolute and relative GET scores at a turbine’s location and the bird’s location and the quality of the golden eagle habitat. In general, if both the turbine and surrounding habitat had high GET scores a bird would be closer to a turbine but this was mediated by the turbine’s status, although not in a consistent way. This is reflected in the much higher share of explained variance associated the turbine ID random factor (Table 2).

**Fig 3:**
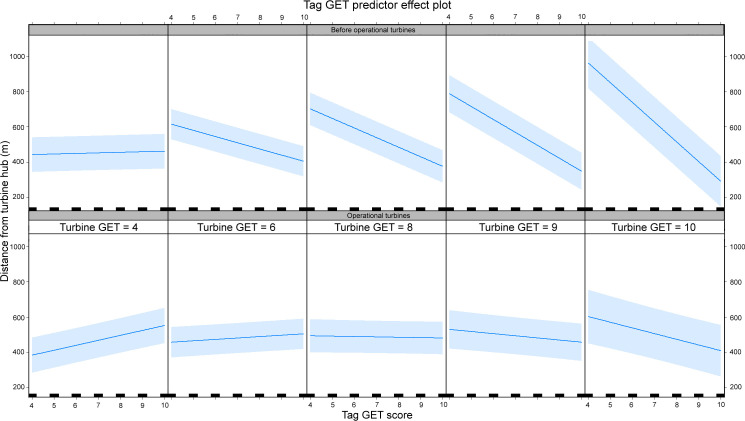
Tag GET effect plot showing the relationship between the fitted distance to the nearest turbine hub (y) and the GET score at a bird’s location. The upper row shows the relationships before turbines became operational and the lower row shows the relationships after turbines became operational. The panels show how the relationship is conditional on the GET score at the nearest turbine’s location. Blue lines and blue shading show the mean and 95% CL ranges, respectively.

The signs (+ or -) of the turbine GET fixed factor in Table 2 are misleading when compared with the fitted and observed relationships (Fig 2). This is probably a consequence of multicollinearity involving both fixed and random factors, but particularly the GET scores and the turbine status (see later after-operation analysis). We investigated this by examining the tag GET predictor effect plots (Fig 3). This shows the complex relationship between the attractiveness of turbine and landscape habitat as quantified by the GET score and how this changed once turbines become operational (lower panel row) compared with before there were turbines at those locations (upper panel row). When the turbine GET score was less than six (generally little used habitat) birds were recorded further from turbines as the GET score at the bird’s location increased. This reflected a situation in which the better habitat was away from the turbines, so birds would be drawn away from the turbine locations. However, the upper panel row (Fig 3) illustrates that, before the presence of operational turbines, birds approached closer as the turbine GET score increased, particularly when the bird was also over more-preferred habitat. Note the increasing steepness of relationships in plots going to the right, illustrating the increasing habitat attractiveness via GET score at the turbine location relative to the attractiveness underlying the bird’s location (Tag GET score). This reflects a situation in which an increasingly attractive turbine location resided in regions of varying attractiveness to eagles. The lower panel row (Fig 3) shows the same relationships were present at the same locations after turbines were operational. This suggests that the attractiveness of turbine habitat can be suppressed once turbines were operational; while there remains, albeit much diminished, attractiveness at turbine locations in the most highly preferred turbine GET scores (9 and 10).

**Fig 4:**
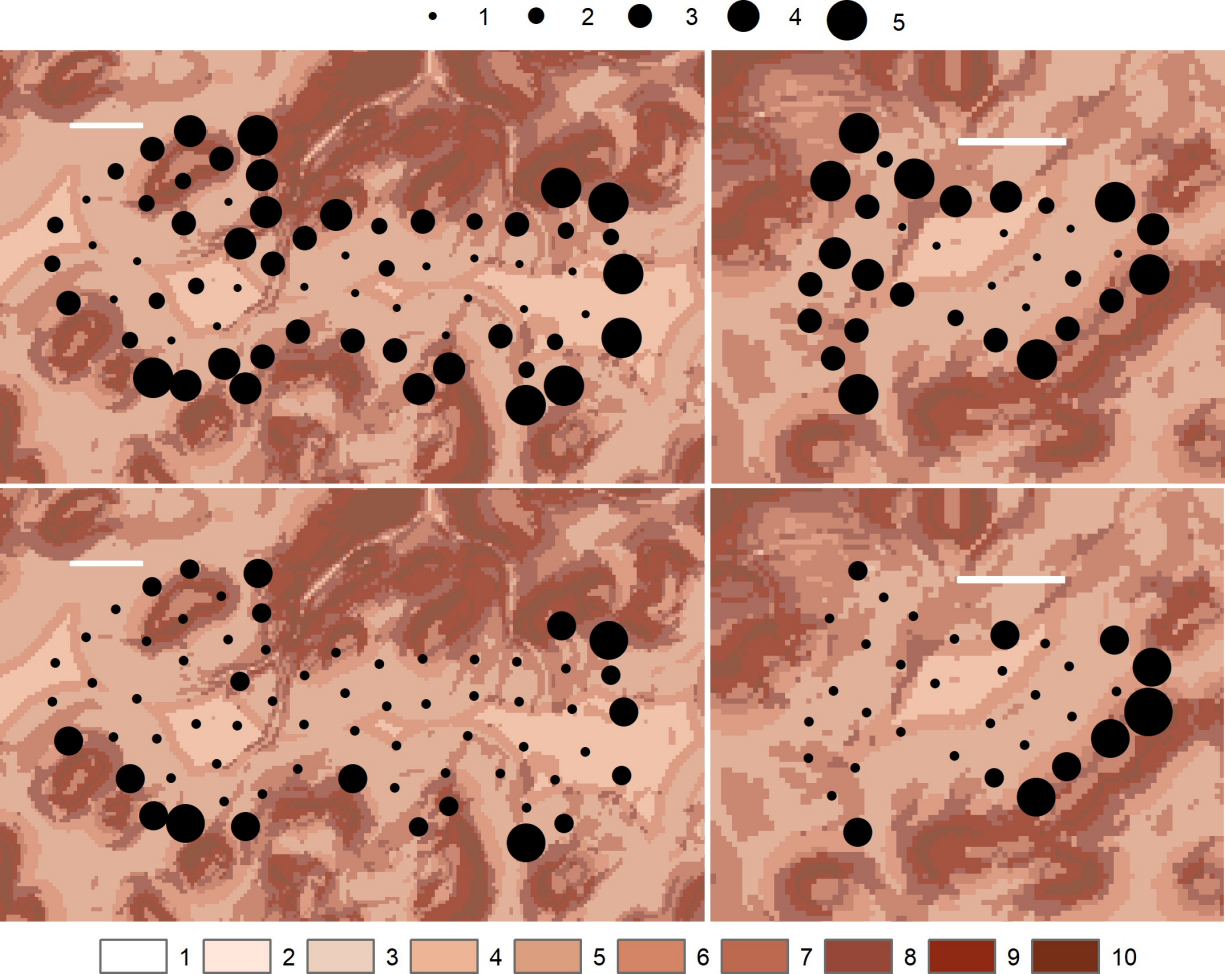
Proportions of eagle tag records at turbine hub locations at Stronelairg (left two panels) and Dunmaglass (right two panels), before and after turbine operation (above and below; by wind farm). Black circles are quantiles (1–5) with larger circles indicating greater proportions of records. The backdrop (1–10) shows the GET score [45] by 50-m pixels in intrinsic habitat preference with a higher score (darker) indicating higher preference. The horizontal white bar is 1 km. Note the relative reduction of records in proximity to ‘inner’ turbine locations after turbine operation and that after turbine operation, golden eagle activity was further restricted to the vicinity of a few ‘outer’ turbines’ locations: Particularly pronounced at Dunmaglass. Contains Ordnance Survey data © Crown copyright and database right 2017.

It is clear from the analysis that much of the variation in approach distances was a turbine-specific issue some of which may have been related to the position of a turbine in a wind farm and the layout of the wind farm and its relationship with surrounding habitat. While we could not incorporate an ‘internal/external’ wind farm turbine factor without risk of spurious results (see above) it was apparent from simple comparisons of use of turbine locations before and after operation date that the operation of turbines effectively caused an abandonment of habitat within the interior of both wind farms (Fig 4). This abandonment seemed regardless of intrinsic habitat preference (GET scores) underlying inner turbine locations. Such that, after turbine operation eagle activity was largely restricted to the vicinity of outer turbines in preferred habitat, and in particular to some outer turbines, often with extensive preferred surrounding habitat (Fig 4).

### After-operation analyses

**Fig 5:**
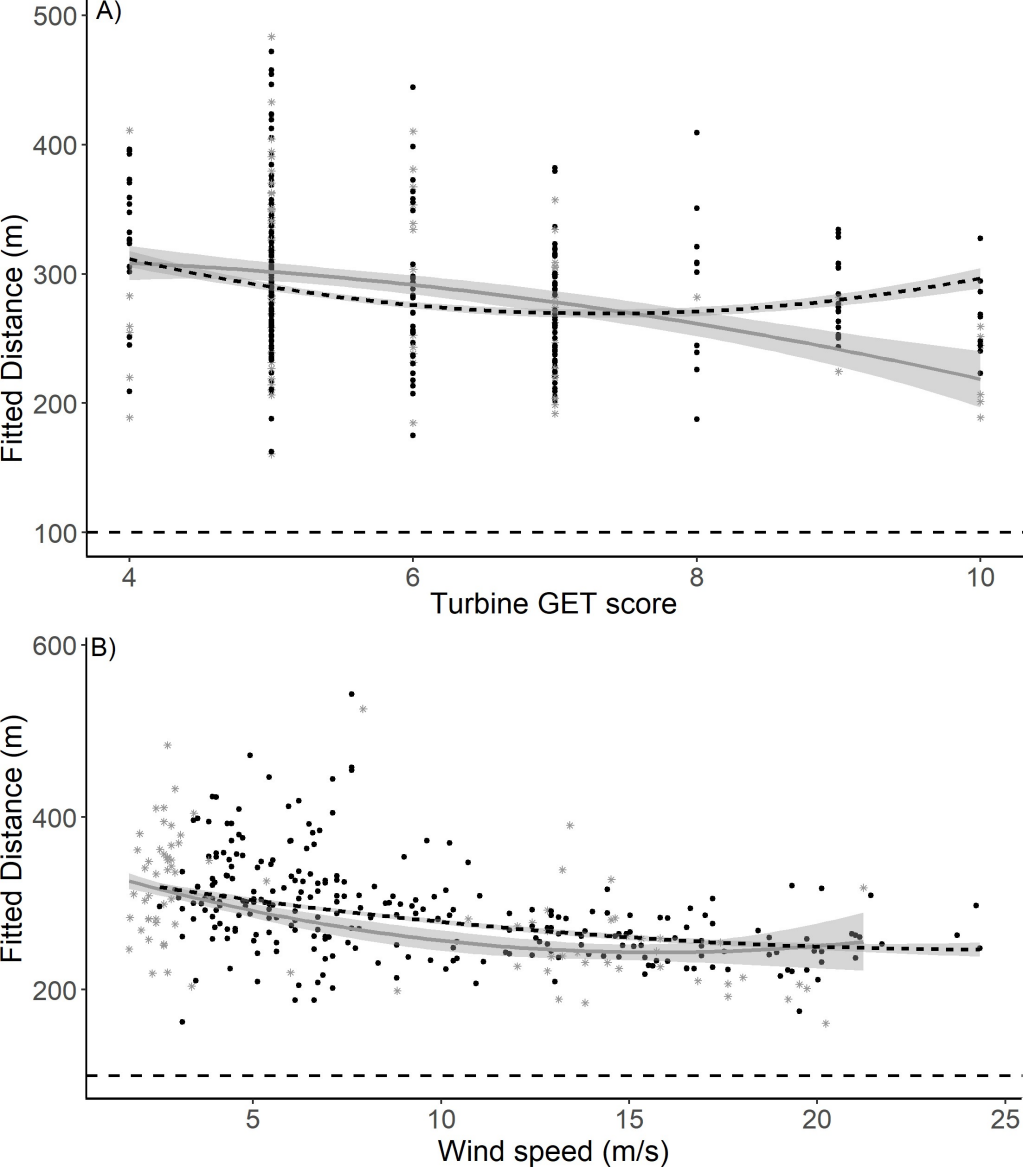
Relationships according to the best after-operation GLMM (Model 24: Tables 3 and 4) between: a) GET score (habitat preference increasing with score) at a turbine and fitted distance of an eagle flight line to a turbine hub, for turbines with moving blades (black dots, black short-dashed mean trend line) or stationary (grey stars, solid grey mean trend line); and b) Wind speed and fitted distance of an eagle flight line to a turbine hub, for turbines with moving blades (black dots, black short-dashed mean trend line) or still (stationary) blades (grey stars, solid grey mean trend line): Trend lines are best-fit polynomials (x + x^2^). In both panels (a and b), grey shading shows 95% CL, and the black long-dashed horizontal line shows 100 m distance to a turbine hub, for illustrative context. Blade lengths from hubs were 57.5 m (Stronelairg) and 40 m (Dunmaglass). No turbine location had a GET score < 4.

**Fig 6:**
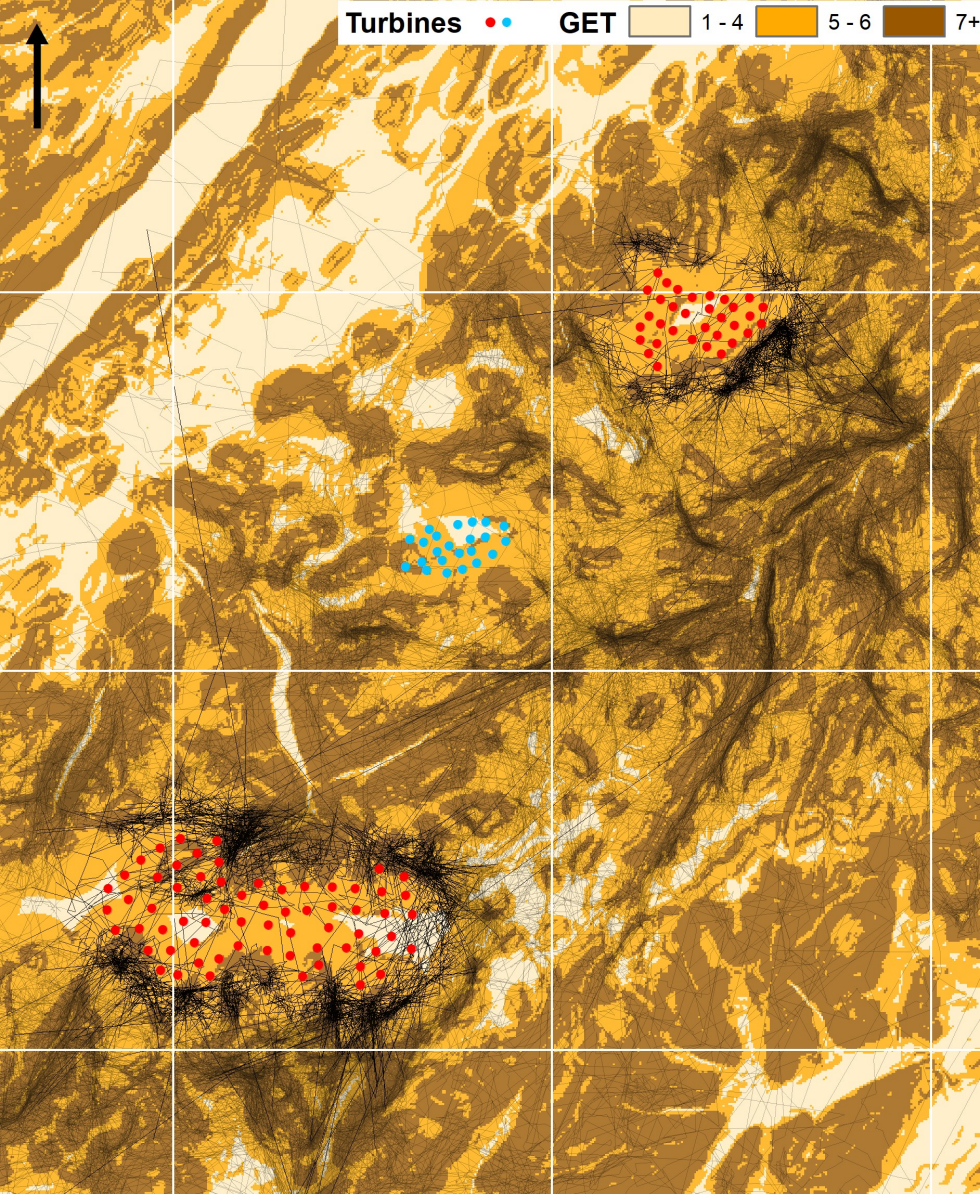
Flight lines derived from GPS/GSM-telemetry data after Stronelairg (lower array) had become fully operational in 2018 (see main text), with a darker emphasis on those within 1 km of the two study wind farms. Turbine locations are red (Stronelairg and Dunmaglass: study sites) or blue (Corriegarth) circles. The white overlay is 10 km grid square with backdrop of GET model predictions of habitat preference [45] in three classes of preference scores (see legend): arrow shows north. Note that in 3-D approximately 75% of flight lines passing ‘across’ the wind farms were at altitudes above the limits of the highest blade tip extension of turbines and <1% were within a rotor blade width of the hub. Contains Ordnance Survey data © Crown copyright and database right 2017.

After-operation analyses used only telemetry data after turbines became operational. These data were different in their derivation to those used in before-and-after-operation analyses by utilising the higher temporal frequency provided by GPS/GSM tags to approximate the closest 3-D proximity of a flight line to a turbine hub. Such approximation should allow a smaller distance than from either the line’s start or end point (Methods). Despite this, it is worth noting that flight line records were again rarely close to operational turbines (Fig 5). This was apparent at both the study wind farms and, subjectively, at a third wind farm located between them (Fig 6: see also Fig 1). The 0.025 quantiles for the calculated distances to a turbine hub were 204 m (still) and 140 m (turning) at Dunmaglass and 124 m (still) and 123 m (turning) at Stronelairg. Thus, birds rarely approached closer than 80 m to the tip of turbine blades.

There were two high-ranking models according to ΔAIC and ΔBIC [114,118]: Models 24 and 26 (Table 3). Model 26 was the most complex and had the lowest AIC score but no significant main effects or interactions. Given the relative merits of AIC and BIC in the circumstances, and to facilitate interpretation and parsimony, Model 24 was selected as the best (Table 3). No problems were apparent with the residuals. 55.9% of the variance was explained by the best model (Table 3); 8.6% was the due to the fixed factors and 47.3% was due to random factors. Although the fixed factors explained a larger proportion of the variance than in the before-and-after model it was still small compared with the effects of the identity of the eagles, turbine identity and the wind farm. The largest component of the explained variance was again the turbine identity random factor (75.2%).

**Table 3:** The twenty seven model candidates in after-operation GLMMs with resultant model selection values of ΔAIC and ΔBIC.

Model	Fixed factors	df	ΔAIC	ΔBIC
0	Null	5	187.1	108.4
1	GET score at flight line (GETtag)	6	185.2	112.0
2	GET score at turbine (GETturb)	6	182.8	109.6
3	Blade motion status (Turn)	6	164.2	90.9
4	Wind	6	105.0	31.8
5	GETturb + GETtag	7	180.4	112.5
6	GETturb + GETtag +GETturb * GETtag	8	158.3	95.9
7	GETturb + Turning	7	160.1	92.3
8	GETturb + Turning + GETturb * Turning	8	137.2	74.8
9	Turning + GETtag	7	162.5	94.7
10	Turning + GETtag + Turning * GETtag	8	156.1	93.7
11	Wind + GETtag	7	103.7	35.8
12	Wind + GETtag + Wind * GETtag	8	106.0	43.6
13	Wind + GETturb	7	101.1	33.3
14	Wind + GETturb + Wind * GETturb	8	103.1	40.7
15	Wind + Turning	7	94.2	26.4
16	Wind + Turning + Wind * Turning	8	69.5	7.1
17	GETturb + Turning + GETtag	8	158.0	95.6
18	GETturb * Turning * GETtag	12	109.6	69.0
19	Wind + GETtag + Turning	8	92.9	30.5
20	Wind + GETtag + Turning + Wind * GETtag +Wind * Turning + GETtag * Turning + Wind * GETtag * Turning	12	61.8	21.2
21	Wind + GETturb + GETtag	8	99.3	36.9
22	Wind + GETturb + GETtag + Wind * GETturb +Wind * GETtag + GETturb * GETtag + Wind * GETturb * GETtag	12	66.3	25.7
23	Wind + GETturb + Turning	8	90.4	28.0
24	Wind + GETturb + Turning + Wind * GETturb + Wind * Turning + GETturb * Turning + Wind * GETturb * Turning	12	40.7	0
25	Wind + GETtag + GETturb + Turning	9	88.7	31.7
26	Wind + GETtag + GETturb + Turning + Wind*GETtag + Wind*GETturb + Wind*Turning + GETtag*GETturb + GETtag*Turning + GETturb*Turning + Wind*GETtag*GETturb + Wind*GETtag*Turning + Wind*GETturb*Turning + GETtag*GETturb*Turning + Wind*GETtag*GETturb*Turning	20	0	2.7

* indicates interaction.

All single and two-way interaction terms in the best model were significant (Table 4). Although significant, the effect of Turn was small with an overall fitted difference of only 5 m (mean distance to turbine hub: 287 m (still) & 282 m (turning)). The observed means were 288 m (still) and 282 m (turning). Overall, birds got closer as wind speed increased (Fig 5) but there was evidence for a possible non-linear relationship when turbine rotors were still. Unlike the before-after analysis, the GET coefficient was negative as expected from the observed distances. Removing the turbine operational status appeared to remove the multicollinearity issues of the before-after analysis.

**Table 4:** Summary statistics for the best GLMM (Model 22: Table 3) examining 3-D distance of eagle flight line records to the vicinity of a turbine location in after-operation analyses: Turbine GET = GET score at the turbine location, Wind speed = mean wind speed at the turbine hub in a 3 hour period (m s^-1^), turning indicates if the turbine blade was still or turning at the time of a bird record.

	Null Model		Full Model	
*Coefficient*	*Estimates*	*P-Value*	*Estimates*	*P-Value*
Intercept	301.24 (265.69 – 336.80)	<0.001	556.39 (435.04 – 677.74)	<0.001
Turning			-183.77 (-292.62 – -74.91)	0.001
Windspeed			-15.02 (-22.82 – -7.22)	<0.001
Turbine GET			-31.70 (-51.24 – -12.16)	0.001
Wind speed x Turning			10.96 (2.44 – 19.48)	0.012
Turbine GET x Turning			23.83 (6.16 – 41.51)	0.008
Turbine GET x Wind speed			1.25 (0.03 – 2.47)	0.045
Wind speed x Turbine GET x Turning			-1.19 (-2.51 – 0.13)	0.076
**Random Effects**				
σ^2^	6,843.1		6,449.3	
τ_00_	5,141.9 _T_ID_		6,145.4 _T_ID_	
	809.7 _ID_		503.0 _ID_	
	234.4 _windfarm_		260.1 _windfarm_	
N	2 _windfarm_		2 _windfarm_	
	84 _turbine_		84 _turbine_	
	8 _ID_		8 _ID_	
Observations	1,704		1,704	
Marginal R^2^/Conditional R^2^	0.000/0.475		0.086/0.559	

Wind farm identity, turbine identity (T_ID) and bird identity (ID) were random factors. 95% CI are given below the estimates. The lower part of the table is information on the random effects. The R^2^ values are marginal (variance of the fixed effects) and conditional R-squared (variance of the fixed and random effects) statistics, based on [117].

**Fig 7:**
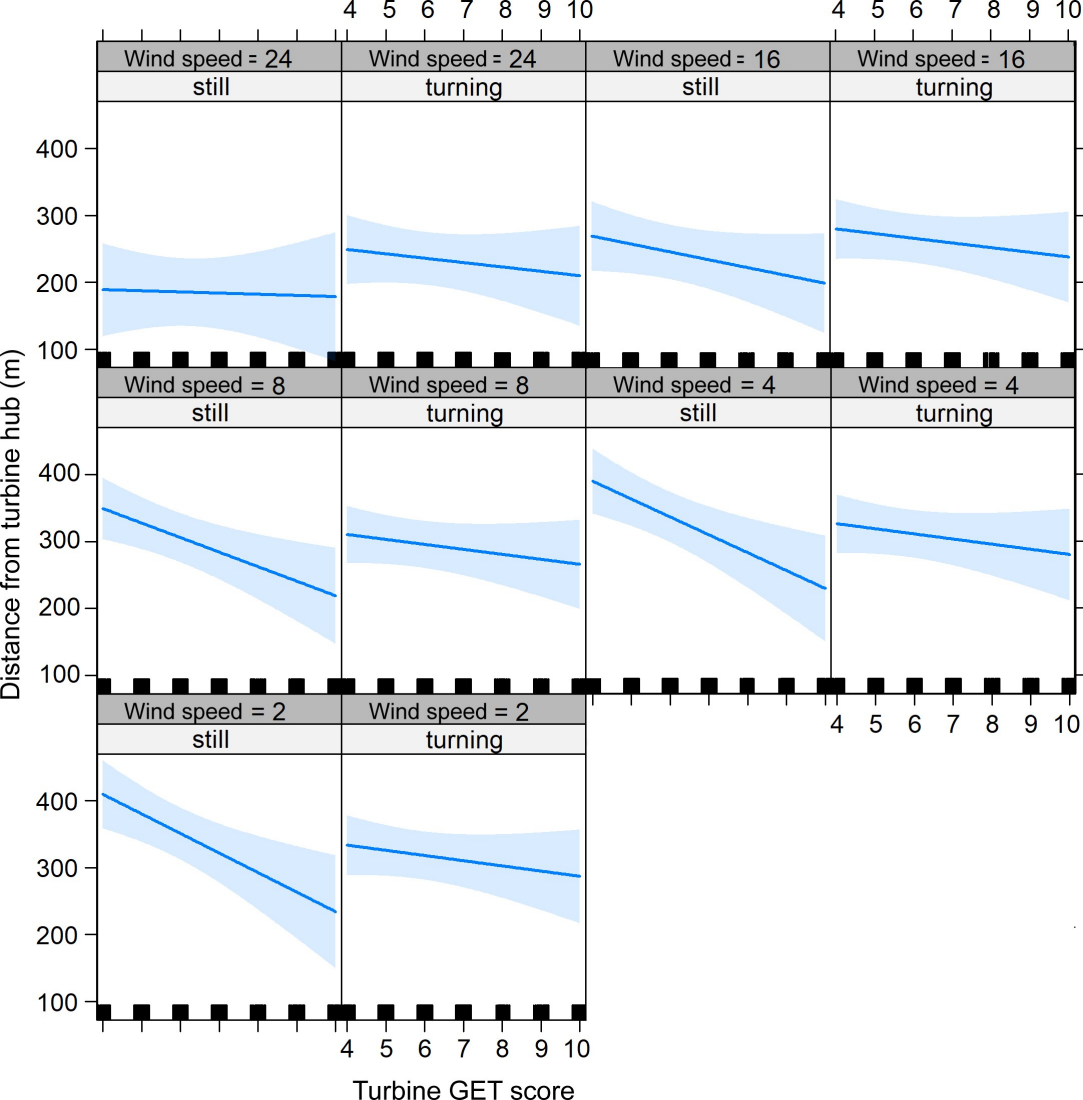
Turbine GET effect plot showing the relationship between the fitted distance to the nearest turbine hub (y) and the GET score at a turbine’s location. The panels show how the relationship was conditional on wind speed (m s^-1^) and whether the turbine blades were still or turning. Blue lines and blue shading show the mean and 95% CL ranges, respectively.

As in the before-after analyses birds got closer as the turbine GET score increased but the distance was conditional on turbine rotor motion and wind speed (Figs 5 & 7). The predictor effect plots (Fig 7) show the general tendency for closer approaches as turbine GET score increased but they also show clear differences depending on whether the rotor was turning or still. The slope of the influence of the turbine GET score was always less when rotors were turning, irrespective of wind speed. However, all slopes increased as wind speed decreased although the closest approaches were, on average, when wind speed was higher.

## Discussion

Results supported our first hypothesis that golden eagles in Scotland showed avoidance of turbines, in keeping with previous studies [56,57,88,89]. After operation of the 99 turbines in the two wind farms, the distance of GPS-tagged eagles to turbine locations increased and turbine operation (Postop) was a significant factor in our best before-and-after-operation model (Table 2).

Displacement from operational turbines through avoidance was not, however, a simple relationship. Modelling indicated that birds’ proximity to turbines–while typically rarely close—was dependent on the intrinsic habitat attractiveness of turbine locations, and its connectivity to the surrounding habitat’s attractiveness where a bird was. Birds were recorded closer to turbines which were in preferred habitat and–especially–when the birds were using nearby preferred habitat. The Postop displacement effect was conditional on turbine-specific habitat preference, as evidenced by the dominating effect of turbine ID in accounting for the explained variances. This is likely to be associated with the strongly influential habitat preference profile of landscape surrounding the turbines and a turbine’s position within the wind farm.

A focus on displacement distances has direct practical application in assessing the impact of the avoidance effect when avoidance equates to functional habitat loss [1,2,5]. We could not explicitly examine the effects of a turbine’s location within a wind farm because modelling birds’ nearest distances to inner turbines is constrained by inter-turbine spacing. Nevertheless, eagles appeared to abandon the vicinity of internal turbine locations after a wind farm became operational (Fig 4). In application, therefore, our results suggested that assessing the impact of a wind farm (in Scotland, at least) should be based primarily on the functional loss of all habitat within a buffer around the outer turbines. A displacement distance of 75 m has been found [57] although on a precautionary basis the buffer should be larger (Figs 2 and 5).

Such an assessment prescription for wind farm proposals implies that collision risk is not a substantive factor in young Scottish golden eagles, and so anticipating population impacts of wind farms should be based on habitat loss and not additional mortality. Although these impacts are substantially different, both are potentially serious [1–3,5,32,77]. As previously noted (Introduction), the antagonism between avoidance and collision risk does not necessarily indicate mutual exclusivity. In at least two studies which concluded avoidance as the substantial response of raptors to wind farms, a few collision fatalities were evident [4,5]. Indeed, consistent with earlier emphases on the importance of individual turbines in generating collision fatalities [6,37,48], we also found that displacement was weakest (and so collision risk strongest) at particular turbines (e.g. Fig 4).

Moreover, at Stronelairg a collision casualty of an untagged subadult golden eagle was found under an outer turbine in early May 2020. Although this might be considered an outlier event in a Scottish context it illustrates further that the two potential adverse effects are not mutually exclusive; even though such collisions are apparently very rare at Scottish wind farms [57]. Such rarity is consistent with the findings of avoidance from both the national study [57] and the present case study.

While collision risk was negligible our results suggest that greatest attention in siting turbines should be on outer locations, such that highly preferred habitat is avoided by developers. This would also help to minimise functional habitat loss, especially if there is a swathe of preferred habitat adjacent or nearby (see also [119]). Our results also suggested that inner turbines, even in preferred habitat, are less risky for collision, although clearly post-operation abandonment of such locations should factor into estimating functional habitat loss. In identifying preferred habitat for golden eagles our study gave additional independent support to the GET model’s predictive capacity [46]. Further, on wind farm design, wind farms composed of single strings/rows are probably worse for elevating collision risk because all turbines potentially contribute to that risk, reflecting findings at two wind farms elsewhere in Scotland [88] and echoed elsewhere [47,48,54,55].

Our after-operation analyses used flight line data which should provide records at greater proximity to turbines than their start and end points (Methods), as the distance can never be greater than the end point distances. These results re-affirmed, however, that eagles rarely went close to operational turbines, consistent with avoidance (Figs 2, 5 and 6). After-operation analyses confirmed the role of the attractiveness of habitat in turbine proximity and also re-emphasised, via the importance of the turbine ID contribution to the explained variance, that birds’ approach-distances to turbines did not have a simple basis.

Under hypothesis 2 (Introduction), we expected that birds would not react differently to rotor blades according to their motion status. Although Turn was a significant fixed effect (Table 4) the overall magnitude of the effect was small. At low wind speeds eagles were recorded further from turbines with motionless blades in less attractive habitat. In practice, this result was moot in application since eagles were recorded at greatest distances from ‘unattractive’ turbines at low wind speed (i.e. they ventured into the wind farms’ immediate surroundings less often), and all turbines’ rotors had an automatic shut-down on blade motion at wind speeds less than c. 3–5 m/s. On the other hand, at higher wind speeds (without automatic rotor shut-down) eagles were recorded closer to turbines with motionless blades in the most preferred habitat (Figs 5A and 7). This implied that when eagles approached turbines more closely, they were aware and less wary of turbines whose blades were motionless.

In practice, through overarching avoidance, birds’ approach distances typically remained large and motionless rotor blades were uncommon at higher wind speeds (when they were stationary at higher speeds, it was usually through routine maintenance and/or a failed rotor). Therefore, the use of a TSS or “Shutdown on Demand” system [3,35] to mitigate avoidance would probably offer little benefit in our study system; especially at inner turbine locations where blade motion status probably had little bearing. Also, there is an important difference between a targeted turbine shutdown as a bird approaches and turbines that have stopped for other reasons. Our turbine shutdown events were not directly comparable to a TSS. In a TSS the turbine is stopped by an intervention which implies it was turning and there was a reasonable wind speed. In many of our studied examples turbines were inoperable because the wind speed was too low; in other cases, the turbines may have been undergoing maintenance. In our data a turbine was likely to have been stationary before a bird approached and birds may respond differently to one that was moving but which then stopped through TSS as the bird(s) approached.

Our study involved a population of facultative soaring birds largely dependent on orographic uplift for external wind energy support of flight. Hence, the GET model (a topographic surrogate for orographic wind uplift resources) and wind speed were highly influential factors in our study. In other situations where anabatic (thermal) uplift is more important–such as for many soaring birds on migration (e.g. [44,45,87])–the value of a TSS for those birds which show avoidance [5,25] may be greater. Given that avoidance and collision risk may not be mutually exclusive such TSSs could have dual benefits.

Under hypothesis 3 (Introduction) we expected that greater wind speed would decrease birds’ distance to turbines. Our results confirmed this expectation. The expectation was because eagles should be further from turbines at lower wind speeds since gaining access to turbines’ relatively high elevations was conditional on the uplift energy provided by wind (in its orographic interaction with topography). Our results reveal that avoidance–as well as our other documented influences—was proximately dependent on wind speed with lower displacement at higher wind speeds. Consequently, while collision risk remained unlikely, because of the distances to turbines, it was greater at higher wind speeds.

Our findings on wind speed again affirms the basis of the GET model, and further confirms that preferred habitat at and/or in the vicinity of outer turbines encourages greater possibility of collision; even if, in our study system, that risk was small. Our results’ support for hypothesis 3 was seemingly in contradiction to an alternative, posited from griffon vulture *Gyps fulvus* studies [37,48]. The differences, however, are explicable by differences in species and study systems and reveal that case studies which focus on detailed data provide insight beyond consideration of wind farms as homogenous entities. Our studies suggest that in Scotland collision risk for golden eagles is not a serious threat; from other research, that risk may be greater elsewhere. A universal application of our results, nevertheless, is that assuming raptor flight behaviour around or in wind farms is random (e.g. [15]) is wrong: on this our study adds to a growing chorus.

Golden eagle collision fatalities are empirically more likely in some other populations [8,10,73,120], although in others the predominance of collision risk is more an assumption [71,121] which may not hold [72] and migrating eagles may show altitudinal avoidance [43]. It has been argued [57] that understanding the balance between the two processes (avoidance and collision) in large raptors is in genotypic predisposition of wariness to the human “super-predator” and phenotypic cost-benefits in its expression. We also found that eagles were typically wary of turbines (with apparent individual differences) and went closer to operational turbine locations which were more attractive through the surrounding habitat preference, consistent with [57]. The balance between avoidance and collision risk is thereby complex [2,3,10,77]; it is demonstrably not simply ‘either/or’, and it is increasingly apparent that it rests on both ultimate drivers and several proximate influences.

## Supporting information

S1 File GPS-telemetry records (before-after analyses). (TXT)

S2 File GPS-telemetry records (turbine turning analyses, post-operational analyses). (TXT)
